# Recreating Tumour Complexity in a Dish: Organoid Models to Study Liver Cancer Cells and their Extracellular Environment

**DOI:** 10.3390/cancers11111706

**Published:** 2019-11-01

**Authors:** Gilles S. van Tienderen, Bas Groot Koerkamp, Jan N. M. IJzermans, Luc J. W. van der Laan, Monique M. A. Verstegen

**Affiliations:** Department of Surgery, Erasmus MC-University Medical Center Rotterdam, 3015 GD Rotterdam, The Netherlands; g.vantienderen@erasmusmc.nl (G.S.v.T.); b.grootkoerkamp@erasmusmc.nl (B.G.K.); j.ijzermans@erasmusmc.nl (J.N.M.I.); l.vanderlaan@erasmusmc.nl (L.J.W.v.d.L.)

**Keywords:** tumour organoids, primary liver cancer, extracellular matrix, disease modelling

## Abstract

Primary liver cancer, consisting predominantly of hepatocellular carcinoma (HCC) and cholangiocarcinoma (CCA), remains one of the most lethal malignancies worldwide. This high malignancy is related to the complex and dynamic interactions between tumour cells, stromal cells and the extracellular environment. Novel in vitro models that can recapitulate the tumour are essential in increasing our understanding of liver cancer. Herein, primary liver cancer-derived organoids have opened up new avenues due to their patient-specificity, self-organizing ability and potential recapitulation of many of the tumour properties. Organoids are solely of epithelial origin, but incorporation into co-culture models can enable the investigation of the cellular component of the tumour microenvironment. However, the extracellular component also plays a vital role in cancer progression and representation is lacking within current in vitro models. In this review, organoid technology is discussed in the context of liver cancer models through comparisons to other cell culture systems. In addition, the role of the tumour extracellular environment in primary liver cancer will be highlighted with an emphasis on its importance in in vitro modelling. Converging novel organoid-based models with models incorporating the native tumour microenvironment could lead to experimental models that can better recapitulate liver tumours in vivo.

## 1. Introduction

Primary liver cancer (PLC) is the second leading cause of cancer mortality worldwide, with a steady increase in incidence over the last few years [1,2]. Each year there are 782.000 new cases and 746.000 deaths from PLC due to a poor five-year survival rate of 10.1–16.6%, and this is projected to increase further in the coming years [3,4,5]. PLC compromises a heterogeneous group of tumours, the most common subtypes including hepatocellular carcinoma (HCC) and cholangiocarcinoma (CCA). Each subtype is characterized by vastly different histological features and a wide variety of mutational landscapes [6,7]. HCC accounts for 80–90% of all cases of liver cancer, and it arises most often in a setting of chronic inflammation and fibrosis due to hepatitis B/C infections, alcohol abuse or autoimmune reactions [8,9]. CCA is subdivided into intrahepatic (iCCA), perihilar (pCCA) and distal cholangiocarcinoma (dCCA), based on its location [10]. Each subtype vastly differs in its clinical presentation and associated treatment regimen, and although they are relatively easy to distinguish, the plethora of subtypes in PLC makes it a very complex cancer to tackle [11,12]. dCCA is beyond the scope of this manuscript because it is not PLC.

The highly malignant behaviour of PLC is not solely the consequence of cellular transformation. Recently, studies have shown the vital role of the tumour microenvironment in the initiation, progression, invasion and metastasis of cancer, particularly within the context of PLC [13,14,15]. The tumour microenvironment is composed of a wide variety of stromal cell types, including cancer-associated fibroblasts (CAFs), endothelial cells, immune cells, and the extracellular environment.

The complex interplay between primary tumour cells, stromal cells, and extracellular environment is a limiting factor in the amount of treatment options currently available for PLC. Additionally, the anatomical and functional restrictions of the liver play a role in this as well [16,17]. Particularly the dysregulation of the extracellular environment, consisting of various macromolecules, via abnormal extracellular matrix (ECM) remodelling is an important aspect of cancer progression [18]. Important HCC treatment options currently are resection, liver transplantation, local ablative therapies, chemotherapy, and intra-arterial treatments [19]. However, curative intent can only be offered in 20–25% of patients [20,21]. Treatment options for CCA are similar to HCC, although fewer patients are eligible for liver transplantation [22,23]. Even after curative-intent resection or liver transplantation, recurrence rates remain high with 49–64% [23]. Thus, there is a clear unmet medical need to develop novel systemic and locoregional treatments that are able to effectively combat PLC.

One pertinent cause of the lack of effective treatments in liver cancer is the limited number of reproducible, reliable models that are able to faithfully recapitulate the complex in vivo situation. Robust animal and human models for PLC can aid in understanding the molecular mechanisms behind the pathogenesis of PLC and provide pre-clinical platforms for the development of novel treatments. Mouse disease models and two-dimensional (2D) cell line cultures have historically been important pillars for preclinical development of cancer therapeutics. Although they have enabled deeper insights into cancer biology and tumorigenesis, their characteristics contain inherent limitations towards their overall applicability. Animal models and their relevance in PLC have been extensively reviewed elsewhere [24,25,26,27] and is beyond the scope of this review. 3D culture has emerged as a superior model over conventional 2D culture. In particular, the establishment of adult stem-cell derived organoids have gained popularity, due to their robust long-term cultivation, self-organizing ability and mimicking architecture and function of the representing organ or tumour [28]. Herein, we first evaluate the existing in vitro models of PLC, comparing traditional 2D and 3D cell culture to organoid approaches, with a focus on the limitations and unique aspects of each modality. Building upon this, we highlight the significance of the extracellular environment in the development of PLC, and argue for the potential benefit of incorporating the extracellular environment into future in vitro models.

## 2. Limitations of Current in Vitro Cancer Models

Tumour-based in vitro models have been developed with a varying degree of complexity and a wide range of quantification methods, from reductionist 2D cell line models to full ‘mini-tumours’ including aspects of the tumour microenvironment [29,30,31]. Cancer model development has historically been focused on creating an all-in-one encompassing model, but more recently models have been designed to probe specific segments of cancer development, including migration, invasion, matrix remodeling, intravasation, and angiogenesis [32,33,34,35]. The complexity of the model should be linked to the objectives of the study, for example an intravasation model has to incorporate an appropriate environment with vessel-like structures mimicking the basal membrane of blood or lymphatic vessels. The next sections aim to illustrate the characteristics and strengths and weaknesses of the various types of in vitro models employed for PLC. Particular focus will be on examples of their applications and the careful consideration in selecting the appropriate modality. A comparison of the different models, broadly classified into 2D cell lines, 3D spheroid-based models and organoids, is summarized in Table 1.

### 2.1. Two-Dimensional Cell Line Models

PLC cell lines have been well-characterized, are low-cost maintenance, and can be used for genetic modification, providing relatively robust and reproducible results [36]. Due to their unlimited proliferative capacity, they serve as a platform for high-throughput and simple mechanistic analysis. For example, a wide panel of 25 HCC cell lines was used to investigate the relationships between HCC molecular subtype and specific drug responses [37]. Research with 2D cell lines is still ongoing, with a particular focus on establishing novel cell lines with unique aspects that are not well-represented in previous cell lines (e.g., gemcitabine resistance in iCCA [38] or a highly tumorigenic HCC) [39].

Efforts have been made using cell lines to model patient-specific tumour characteristics, both in terms of phenotype and genotype [40,41]. However, 2D culture is inherently disadvantaged due to its culture conditions on a plastic substrate. This culturing method does not allow for mimicking of the natural structure of the tumour, and lacks representative cell-cell and cell-ECM interactions. These interactions have an important role in a wide variety of cellular activity in PLC, including differentiation, proliferation, gene expression and drug responses [42,43,44]. Secondly, the monolayer culture results in unlimited access to a serum-based medium that can lead to a selection procedure for certain cell types and, thus, another cause for poor translational quality to an in vivo situation. In vivo, serum is lacking and nutrient, oxygen, and metabolite gradients are important for the development of PLC [45,46]. These gradients are non-existent in a 2D culture model. As a consequence of the limitations of 2D systems for modelling PLC, 3D systems were developed as an alternative to better mimic the natural situation.

### 2.2. Three-Dimensional Liver Cancer Models

Culturing cells in a 3D environment leads to different morphological and physiological traits compared to conventional 2D culture [31,43]. This behaviour in 3D is assumed to be more reflective of the in vivo behaviour of the tumour, as it is able to more accurately mimic the native tumour microenvironment. The tempo-spatial arrangement that is present when adding an extra dimension is crucial in this aspect. This has an effect on cell surface receptor interactions with other cells and their environment, and also through the physical constraints now placed on the cells in a 3D environment rather than 2D [43,47,48]. 3D culture has therefore been more widely adopted for cancer research applications, from drug discovery to investigations into cancer cell biology [49,50]. 3D cultures can be broadly divided into two approaches: scaffold-based and scaffold-free.

#### 2.2.1. Scaffold-Based Models

Scaffold-based 3D cultures can be produced via cell seeding on a cellular 3D scaffold or by encapsulation into a liquid solution followed by solidification or polymerization of the liquid into a hydrogel. Both synthetic and natural materials are used in PLC research, either singular or as a combination, including polyvinyl alcohol (PVA)-gelatin [51], matrigel [52], chitosan-alginate [53] and collagen [54]. Utilizing an alginate-based scaffold with multiple HCC cell lines, Takai et al. [55] showed that a 3D environment results in better mimicking of glandular epithelium and showed more resistance to chemotherapeutic agents compared to 2D. The increased resistance to (chemo)therapeutic agents in 3D compared to 2D is a widespread phenomenon within HCC culture systems [53,54,56], because they better mimic the in vivo situation. Similar findings are present for 3D CCA cultures [57,58]. A current issue with scaffold-based models for PLC is the wide variety of (bio)materials and cell sources that are utilized, making direct comparisons between the effectiveness and biological relevance strenuous, thus hindering reproducibility. Creating a more systematic approach for spheroid tumour creation, for example through a pre-selection for spheroids with homogenous shape and volume [59], is a possible solution to the cell source variability issue. Additionally, comparative studies on materials that are appropriate for PLC mimicking could provide more insight into the material of choice.

#### 2.2.2. Scaffold-Free Models

Scaffold-free cultures do not require a base for cells to attach to and are mostly produced with bioreactors to direct single cells into spheroid-like structures, including forced floating, hanging drop, and agitation-based bioreactors [50,60,61]. Spheroid cultures are also more resistant to chemotherapy, possible due to the increased cell-cell interactions and steric hindrance caused by their aggregation [62]. HCC cells cultured within a Rotating Wall Vessel (RWV)-bioreactor were used to screen for metastasis-related genes [63]. The 3D spheroid was required to identify adhesion molecules and other cell-cell interaction-related genes [63], which is not possible with 2D cell culture. Combining scaffold-free and scaffold-based techniques, Tang et al. [64] used a poly (lactic-co-glycolic acid)-scaffold within a RWV-bioreactor to create tumour-like spheroids with high metastatic potential. The 3D spheroid was more closely resembling in vivo metastatic HCC tumours in regards to metastasis-related gene expression, protein secretion, and tumorigenicity compared to 2D models [64]. Nevertheless, this study did not compare the tumour characteristics of the 2D and 3D model to the original patient-specific tumour, but rather to general metastatic HCC tumours.

Thus, 3D cultures using both patient-derived and mutation-induced PLC cell lines, whether scaffold-based or scaffold-free, still exhibit important limitations. These include lack of genetic diversity found in the original tumour after long-term expansion and unavailability of patient-matching healthy control cells.

## 3. Emerging Organoid Models

The term ‘organoid’ has been used for a variety of different culture conditions [65,66], predating back to 1992 for mammary gland cell cultures [67]. For this review, the term organoid will refer to continuously proliferating primary epithelial cells that are embedded in a 3D matrix, specifically driven by Lgr5+ stem cells [68].

### 3.1. Organoid Culture Systems

The establishment of organoid culture is fundamentally based on the notion that there are cells residing within each organ that possess stem cell characteristics and a self-organizing capacity. Various culture systems have been reported for these epithelial cells, but the missing ingredient was the lack of robust long-term culture possibilities. With the discovery of Lgr5 as a marker for these adult stem cells about a decade ago, a novel in vitro system was developed that was able to sustain long-term culture [68]. The embedment into a laminin-rich 3D matrix, Matrigel or Basement Membrane Extract (BME), allows epithelial organoids to form from single Lgr5+ adult stem cells [68]. Through organ-dependent adaptations to the protocol, organoids have since then been established from a multitude of organs, including liver [69], prostate [70], colon [71], lung [72], pancreas, [73] and recently also for human cancers, including PLC [74].

Although organoids possess qualities that are similar to 3D spheroid culture of established cell lines, there are certain fundamental differences between the two culture methods. Firstly, a unique aspect of organoid culture is their ability to be established from patient-specific material and, thus, provide a solid platform for potential precision medicine approaches through direct chemotherapeutic testing or through molecular characterization [75]. Organoids initiated from non-tumour healthy cells from the same patient additionally provide a reliable control for comparative studies to malignant, neoplastic cancer cells. Furthermore, through organoid culture pre-cancerous cell types can be propagated, expanding the different stages of cancer progression that can be investigated in vitro. Lastly, organoids, although relatively complex 3D structures, are highly amendable to a variety of characterization techniques, both molecular and cellular. Furthermore, they can be genetically modified to investigate cancer progression-related mechanisms including, but not limited to, the discovery of cancer driver genes and drug resistance.

### 3.2. Primary Liver Cancer (PLC) Organoids

**Figure 1:**
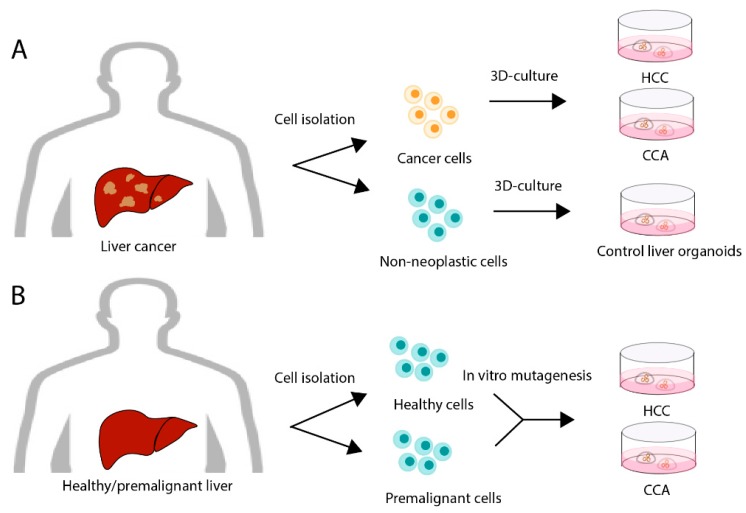
Generation of human primary liver cancer organoids. (**A**) Primary liver cancer organoids can be generated from neoplastic tissue via cell isolation. Paired non-neoplastic tissue can be obtained for control liver-derived organoids of the same patient. (**B**) Alternatively, liver tissue from healthy individuals or patients with liver disease at premalignant stages could be isolated and genetically modified in vitro to create various stages of primary liver cancer development. HCC: Hepatocellular carcinoma; CCA: Cholangiocarcinoma; 3D: Three-dimensional.

Human PLC organoids have been established in several studies [74,76,77] (Figure 1A). The principal study successfully developed organoids representing three major types of PLC: HCC, CCA, and a combined hepatocellular-cholangiocarcinoma (CHC) [74]. Since the differences in PLC subtypes, different media were developed and used for culturing these different organoids. There was a high level of similarity between the histological appearance of the primary tumours and the associated tumour organoids, even after long-term culture [74]. With this in mind, the organoids were used for a variety of applications including prognostic biomarker discovery, drug response studies with a small panel of drugs, and the identification of genes that are associated with a worse prognosis in PLC. Correlations between mutational profiles and drug sensitivities were probed, as well as the discovery of a potential new therapeutic drug, SCH772984, for PLC. In a similar fashion, Nuciforo et al. confirmed the maintenance of morphology, tumour marker expression, and genetic heterogeneity of organoids compared to the originating tumour by obtaining the organoids from tumour needle biopsies [76].

Building upon this, Li et al. expanded drug responses from PLC organoids by using a panel of 129 cancer drugs on HCC and CCA organoid lines, finding that there is a substantial intertumour as well as intratumour heterogeneity present [77], highlighting the importance of using organoids as part of the pre-clinical drug discovery pipeline. To note, the generation efficiency of organoids from HCC patients reported in these studies ranged from 26–47%, and successful generation has not been established for Edmondson grade I and II HCC tumours [74,76]. The next vital step for organoids to become established as a valid pre-clinical platform is to investigate the similarities between patient responses and the responses of the patient-derived organoids to therapeutics. Combining these efforts with the set-up of an organoid biobank, as already underway for colorectal and breast cancer [78,79], will be important next steps in the validation of organoids.

A different approach to study PLC with organoids is through driving healthy organoids towards cancer through engineered genetic mutations (Figure 1B). Via this mechanism, tailor made PLC organoids can be created to examine the effects of certain genetic mutations on the initiation, progression and drug responsiveness of PLC. To this extent, murine liver organoids were transduced with lentiviral vectors to induce Ki-ras (KRAS) activation, known to control cell proliferation and often associated with cancer development [80]. In this study, no single genetic alteration could induce tumour formation, but mutant KRAS with repression of other major tumour suppressor genes co-operating was required to induce tumour development. Additionally, two presumed oncogenes (mutant *Pik3ca* and *FGFR2-AHCYL1* fusion) in iCCA were induced, but showed to be only modest drivers for tumorigenesis in liver-derived organoids, suggesting that additional mutations and/or environmental or epigenetic factors are required.

### 3.3. Current Limitations of Organoid Models

Although organoids are widely regarded as a highly promising personalized in vitro model, several limitations are still present. As PLC contains substantial intertumour and intratumour heterogeneity, at present it is unknown how this cellular heterogeneity is recapitulated in the organoid cultures. Even though PLC organoids presumably are derived from rare cancer stem cell populations residing in tumours, to date it is unclear whether all cancer stem cell clones have similar organoid-initiation potential [74]. Organoids are solely epithelial-sourced and, thus, cannot fully recapitulate the complicated tumour microenvironment. They lack blood vessels, immune cells, CAFs, amongst other relevant cell types. Drug responses and cancer progression are heavily influenced not only by the primary tumour cells themselves but also the complete microenvironment in which these cells thrive. Various studies have focused on combining organoids with the restoration of the immune-component of the TME, including co-cultures with lymphocytes [81] and stromal cells [82], and the combination with immune cells relevant for PLC could aid in the development of novel immunotherapies. These efforts are instrumental in creating a cancer model that faithfully recapitulates the complete tumour.

Concurrently, the predominant protocol of organoid culture requires animal-derived hydrogels, such as Matrigel or BME. Although well-suited for the culture of organoids, finding better matrices is pertinent due to the lack of controlled modifications and undefined growth factors present. Next generations of matrices for organoid culture are being explored, for example a synthetic polyethylene glycol (PEG)-based hydrogel was able to support murine liver organoid expansion to a certain extent [83]. Building upon this, other attempts have successfully managed to create alternative hydrogels that allow for organoid formation and expansion with, in some cases, similar efficiency as Matrigel [84,85].

Converging these last two limitations of tumour organoids show a clear common denominator and underexplored topic: the extracellular environment of the tumour. Shifting the focus towards the extracellular environment allows for investigations into cell-ECM interactions, while at the same time exploring ways to find alternative culturing methods for cancer organoids. Hence, this review will delve more into the critical role the extracellular environment plays in PLC. We will discuss the possibility of converging organoid-based models with systems incorporating models of the tumour microenvironment. In turn, this can lead to a better understanding of the biology behind ECM composition and its influence on carcinogenesis.

## 4. The Liver Cancer Extracellular Environment

The classical view of cancer development through cancer cells accumulating consecutive genetic mutations in a multi-step process has been the predominant outlook for the last decades. Genomic-focused studies have found various important (driver) oncogenes and tumour suppressor genes but with relative modest success. This has led to a more comprehensive view that sees tumour progression as a dynamic crosstalk-driven element that encompasses the complete microenvironment of the tumour [86,87]. Thus, a deeper understanding of these interactions occurring in the tumour microenvironment, herein focused on the tumour extracellular environment (TEE), is required to fully understand biology in both HCC and CCA. This extracellular environment consists of a wide variety of components including macromolecules (i.e., collagen, fibronectin, laminin, elastin), remodelling proteins, matrix metalloproteinases (MMPs), and cytokines, each playing a different role in carcinogenesis [88,89,90]. HCC often develops in already damaged environments containing large areas of inflammation and fibrosis [91], while CCA is often characterized by notable desmoplasia [92]. Important to enhancing our understanding of the tumour environment is to elucidate the contribution of distinct components within the TME towards the tumour progression.

### 4.1. Role of the Extracellular Environment in Hepatocellular Carcinoma

Hepatocellular carcinoma is a fairly unique carcinoma in that fibrosis and chronic inflammation, by hepatitis B and C commonly precedes the development of HCC [93], with up to 90% of HCC cases experiencing fibrogenesis [94]. In general, a pathological hallmark of carcinoma is the abundant deposition of ECM. Multiple cellular sources are responsible for this phenomenon, most prominently CAFs, inflammatory cells, and to some extent primary tumour cells undergoing epithelial-to-mesenchymal transition (EMT) [95]. CAFs are able to efficiently turnover the composition of the matrix by, on the one hand, producing ECM components and, on the other hand, secreting matrix metalloproteinases (MMPs) [96]. These fluctuations in proteomic-content can cause genetically similar cells to exhibit highly differential responses, and this heterogeneity in phenotype has been shown to be important for diagnosis and treatment [97]. Matrix remodelling exhibits itself in a variety of ways, including the distribution of laminins, fibronectin and collagen IV indicating the level of differentiation of the tumour. Particularly, Laminin-322 is important in hepatocellular carcinoma [98,99]. Giannelli et al. showed that the presence of Laminin-5, recently renamed to Laminin-322, was associated with a higher occurrence of metastasis and a worse prognosis in HCC patients [99]. It also plays a prominent role in maintaining quiescence and increasing resistance of the cells residing in the cancer stem cell niche in HCC cases that have hepatic progenitor cell features [100]. More than just singling out the importance of Laminin-322, these studies show that tumour behaviour is highly plastic and dependent on the micro-environmental components for input. Additionally, differentiated tumours show an intact basement membrane whereas undifferentiated tumours show a more defective basement membrane composition, possibly linked to increased invasion and metastasis [101,102]. By using a novel quantum dot-based multiplex imaging technique in combination with conventional immunohistochemistry, Fang et al. showed that the remodelling of the ECM is a continuous process that occurs throughout HCC progression and allows for invasion of HCC cells into the surrounding tissue, particularly through collagen type IV degradation [103]. Furthermore, Zhang et al. [104] used large-scale transcriptome analysis to show that extracellular matrix protein interactions contributed to portal vein tumour thrombus (PVTT), a serious complication of HCC. It is evident from these studies that the composition and presence of specific ECM-related proteins are important in altering cancer cell behaviour in HCC. Particularly EMT is heavily intertwined with a deregulated environment in HCC, as tumour metastasis is prompted by Twist1, a vital regulator of EMT, through subsequent MMP activation [105]. Furthermore, periostin, an EMT protein, has been found to be overexpressed in HCC compared to healthy liver [106].

In addition to composition, the physical properties of the TEE also influences PLC through providing biochemical and mechanical cues to PLC cells, with integrins regulating internal signalling pathways. Lysyl oxidase 2 (LOXL2) modifies the stiffness of the extracellular environment in HCC through cross-linking of fibrillary collagen I, which is involved in tumour growth and metastasis [107]. Subsequently, integrin beta-1 expression was upregulated with an increased mechanical stiffness of the TEE in vivo, and was correlated with Edmondson’s pathological grade, metastasis and Hepatitis B infection [108].

### 4.2. Role of the Extracellular Environment in Cholangiocarcinoma

A prominent desmoplastic extracellular environment is a hallmark of CCA, the bulk consisting of collagen type I and fibronectin [109]. Particularly increased deposition of tenascin is correlated with worse patient outcomes, increased tumour size, and metastasis to the lymph node [110]. Similarly to HCC, periostin is also expressed in stroma and epithelium of CCA, and a prognostic marker for shortened survival, thus showing the potential link with EMT and the extracellular environment in CCA [106]. Modulation of the TEE in CCA is caused by MMP-7 and MMP-9, and their expression is associated with a worse prognosis and lymph node metastasis, respectively [111,112]. The TEE indirectly influences other important processes related to tumour progression, e.g., localized matrix modulation is required for the formation of new blood vessels within the tumour environment [13]. Angiogenesis is necessary for sufficient nutrients and oxygen to support growth of the tumour, and this process is regulated by metalloproteinases and cathepsin B in CCA [13]. Lower survival rates, higher re-occurrence rates and increased metastasis are all linked to angiogenesis in iCCA patients [113]. Possible targeting of the matrix to prevent angiogenesis occurrence could provide novel therapeutic targets for CCA in the future.

Another effect of the desmoplastic TEE is through an increased rigidity resulting from remodelling proteins, particularly LOXL2 in CCA, crosslinking collagens and elastins [114]. Cells respond to different stiffness levels through re-organization of the cytoskeleton, with mechanosensors Yes-associated protein (YAP) and its transcriptional co-activator with PDZ-binding motif (TAZ) playing important roles acting as transducers of mechano-information [115,116,117]. Under healthy circumstances, the rigidity of the ECM provides tumour-suppressor functions [118], but during tumour progression the increased rigidity of the ECM can increase malignant behaviour of tumour cells through stimulating the nuclearization of YAP [119]. As a result, almost all CCA patients exhibit a high expression of YAP and TAZ [120], and increased interest has been taken in strategies targeting YAP. With only limited animal models of CCA available that give attention to YAP/TAZ, a focus on better in vitro models that incorporate the ECM could give more mechanistic insight into its role in CCA progression.

## 5. Adapting Organoid Models to Study Liver Cancer Cell Interactions with the Extracellular Environment

With the vital role the extracellular environment plays in all facets of tumour biology, it is evident that creating an improved in vitro model of tumour performance requires a close mimicking of the composition, morphology and characteristics of the environment. Currently, research into elucidating interactions between the extracellular environment and cancer cells in PLC is relatively scarce. Particularly organoids as a platform for studying cellular interactions with the tumour extracellular environment in the context of PLC is non-existent, thus we look towards alternative culture models to highlight the importance of adapting organoid models to study these interactions. Broguiere et al. [85] used a soft fibrin gel with laminin-111 supplementation to allow for the culture of multiple organoid types, including pancreatic ductal adenocarcinoma (PDAC). This well-defined matrix is the first to show formation and long-term expansion of organoids encompassing progenitor as well as differentiated cells, in a similar fashion to Matrigel. Using this novel culture method, they were able to probe the mechanical forces related to the interactions between intestinal organoids and the environment, finding that the transition from spherical to budding organoids also showed a transition in contractile forces present. Although not directly coupled to tumour biology, this proof of concept shows the possibilities in uncovering tumour organoid interactions with the extracellular environment through exploring alternative culturing methods and probing singular effectors such as specific ECM components.

**Figure 2:**
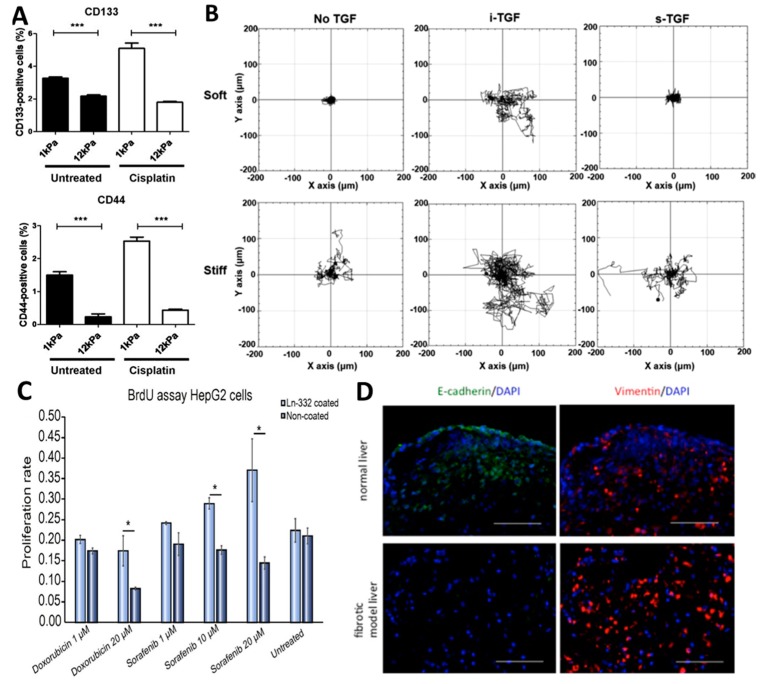
In vitro culture models to highlight the importance of the extracellular environment. (**A**) Flow cytometric analysis of cancer stem cell markers cluster of differentiation 133 (CD133) and cluster of differentiation 44 (CD44) in HepG2 cells cultured on soft or stiff matrices for five days shows effect of matrix and chemotherapy on stem cell marker expression. Adapted with permission [121]. Copyright 2019 American Chemical Society. (**B**) Representative trajectories of HCC cell migrations in different matrices. Adapted with permission [123]. (**C**) Proliferative capacity of coated and non-coated HepG2 cells after doxorubicin or sorafenib treatment for 48h (*n* = 4). Laminin-322 (Ln-332) coating increased chemoresistance of HepG2 cells (scale bars: 100 μm) Adapted with permission [100]. (**D**) Immunofluorescence staining showing the number of vimentin-positive cells is increased and E-cadherin-positive cells is decreased in a fibrotic liver scaffold compared to normal liver, indicating the promotion of EMT phenotype. (scale bars: 100 μm). Adapted with permission [129]. BrdU: 5-bromo-2’deoxyuridine; DAPI: 4′,6-diamidino-2-phenylindole; TGF: transforming growth factor. * *p* < 0.05; *** *p* < 0.001.

In direct relation to PLC, but using cell lines, Schrader et al. [121] showed that an increased matrix stiffness, characteristic of a fibrotic and cirrhotic liver environment, increased the proliferative capacity and chemotherapeutic resistance of HCC cells in vitro (Figure 2A). Interestingly, cancer stem cell characteristics were also influenced by the stiffness of the matrix, with low stiffness resulting in a cell population with increased clonogenic capacity. It is evident that the mechanical characteristics of the environment profoundly impact the phenotype of liver cancer cells in vitro, congruent with in vivo findings [122]. Tang et al. [123] uncovered the interplay between mechanotransduction and biochemical signalling on regulation of cell fates in HCC by using a stiffness-tunable scaffold and TGF-β1 as a biochemical cue. Synergies between matrix stiffness and biochemical signalling was seen through different levels of migratory and invasive activity in HCC cells (Figure 2B). Some aspects of tumour biology are regulated by single aspects of the extracellular environment, while others require the synergy of the encompassing environment and the multiple functions of the environment, and different in vitro models should be utilized depending on the objective. For example, in hepatic progenitor cell (HPC)-related HCCs the presence of laminin-332 in vitro induced a phenotypic switch resulting in a more quiescent cell state, and an increased resistance to doxorubicin and sorafenib [100] (Figure 2C). Chaijan et al. [124] attempted to model invasion of CCA within Matrigel in vitro, by showing that the ECM, in this case Matrigel, promotes invasive outgrowth through L-plastin upregulation. However, Matrigel is considered a poor substitute for true in vivo recapitulation of the TEE, and this was confirmed through the lack of correlation between L-plastin expression in CCA tumours and tumour differentiation and metastatic status [124]. Alternative ECM-mimicking materials that more closely resemble the intricate composition and structure of the TEE could prove fruitful for these applications, however, engineering highly complicated structures is a difficult challenge. Decellularization, a technique used to isolate the extracellular matrix of a tissue, is a promising top-down approach to study the extracellular environment that can complement the more bottom-up approaches currently utilized. Through harnessing the body’s ability to create complex structures, specific features of the tumour environment remain intact. Although more widely adopted in (healthy) tissue engineering strategies for organs including heart [125], kidney [126], cartilage [127], and liver [128], decellularization could also play a role in unravelling tumour biology and matrix-cell interactions. Miyauchi et al. [129] used decellularized fibrotic rat livers to create a native-like scaffold for HCC cells. Compared to a healthy decellularized liver scaffold, HCC cells showed an increased induction of EMT phenotype, increased proliferation and increased chemoresistance, all vital ingredients for cancer progression in vivo (Figure 2D). This culture system is optimally suited to explore the biological behaviour of cancer cells within natural fibrotic or healthy environments. Recently, Salim et al. [130] were able to decellularize and compare healthy, fibrotic and hepatocellular carcinoma rat liver samples on a primarily histological basis. Although the study design was basic and lacking any type of recellularization, it paves the path for more research into the use of decellularized scaffolds as a basis for in vitro PLC research. The usage of rat-derived livers in both previous studies is an important limitation of this technique. Additionally, this top-down approach is not suitable for studying singular aspects or components of the TEE, thus, should be seen as complementary to other extracellular matrix mimicking techniques.

## 6. Conclusions

The ideal in vitro pre-clinical PLC model would eliminate species differences encountered in in vivo mouse models, and be able to accurately recapitulate the in vivo tumour of each individual patient, and allow for (direct) drug response testing. To this extent, the emergence of organoids in PLC has been highly promising as a physiologically relevant in vitro model. The fact that organoids can be established with high efficiency from patient-derived tissues, can represent the diverse spectrum of cancer subtypes, and can be used for both basic and translational research shows the high potential and versatility of this platform. In the future, it is vital to confirm the currently anecdotal validation, mainly in cystic fibrosis, of organoids as diagnostic tools and obtain robust specificity and sensitivity data on drug responses in organoids derived from individual patient’s tumours. Recent results with gastrointestinal organoids in a clinical setting have been hopeful [131]. Well-designed prospective clinical trials on transcriptome, genome and biochemical analysis on organoids for correct stratification of PLC subtypes could result in organoids profiling becoming a mainstay in both preclinical drug discovery and personalized guidance on future treatment of cancer patients.

However, the ideal in vitro model should also include the incorporation of the tumour microenvironment. Efforts are underway by co-culturing cancer organoids with cellular components of the microenvironment, but the dependence on a basement membrane-like matrix is a hindrance for revealing the full potential of tumour organoids. Thus, incorporation of improved extracellular matrix mimicking substrates into a physiologically relevant in vitro system for experimental liver cancer models could increase our understanding of the biology behind tumour extracellular matrix composition and its influence on cancer. Converging novel organoid-based models with systems incorporating the native tumour microenvironment could lead to experimental models that can better recapitulate liver tumours in vivo.

## Figures and Tables

**Table 1:** Key differences when comparing cellular characteristics and applications in two-dimensional (2D) cell lines, three-dimensional spheroids and organoids.

Comparison	2D Cell Lines	Spheroids	Organoids
**Origin of cells**	Immortalized cell lines	Often immortalized cell lines or tumour biopsies	Tissue-specific stem cells
**Morphology**	Sheet-like flat monolayer	Cell-clusters within 3D environment	Self-organizing, mimicking organ structure
**Drug sensitivity**	Very effective due to 2D morphology	More resistant compared to 2D, better in vivo drug response predictor	Patient-specific responses and matched controls for personalized therapy and best in vivo drug response predictor
**Throughput drug screening**	Suitable for high-throughput screening	Less suitable for high-throughput drug screening	Less suitable for high-throughput drug screening
**Resource costs**	Low	Medium	Medium-high
**Heterogeneity**	Cell-line derived from a single cell	Related to initial cell population	Related to patient used for cell isolation
**In vivo features**	Cellular properties	Cell-cell interactions, hypoxia, drug penetration, production of ECM	Cell-cell interactions, mutational landscape of original tumour, cellular heterogeneity
**Long-term expansion**	Immortalized for easy expansion	In few cases long-term reported, loss of heterogeneity	Robust long-term expansion with maintenance of heterogeneity
**(Bio)materials for culture**	Plastic or biomaterial coatings	Wide variety of (bio) materials	Primarily Matrigel/BME

ECM: Extracellular matrix; BME: Basement Membrane Extract.

## Abbreviations

PLC	Primary liver cancer
CCA	Cholangiocarcinoma
iCCA	Intrahepatic cholangiocarcinoma
pCCA	Perihilar cholangiocarcinoma
dCCA	Distal cholangiocarcinoma
CAF	Cancer-associated fibroblast
ECM	Extracellular matrix
PVA	Poly(vinyl alcohol)
RWV	Rotary wall vessel
LGR5	Leucine-rich repeat-containing G-protein coupled receptor 5
KRAS	Ki-ras
Pik3ca	Phosphatidylinositol-4,5-bisphosphate 3-kinase, catalytic subunit alpha
FGFR2-AHCYL1	Fibroblast growth factor receptor 2-adenosylhomocysteinase like 1
PEG	Polyethylene glycol
MMP	Matrix metallopeptidases
TME	Tumour microenvironment
PVTT	Portal vein tumour thrombus
EMT	Epithelial-to-mesenchymal transition
TEE	Tumour extracellular environment
LOXL2	Lysyl oxidase like 2
YAP	Yes-associated protein
TAZ	Transcriptional co-activator with PDZ-binding motif
PDAC	Pancreatic ductal adenocarcinoma
